# Supplementary material for: Resolving the full spectrum of human genome variation using Linked-Reads
Source: Genome Res. 2019 Apr;29(4):635–45. doi: 10.1101/gr.234443.118 (PMC6442396; doi:10.1101/gr.234443.118)
Supplement: Supplemental Material [file supp_gr.234443.118_Supplemental_longranger-2.2.zip › longranger-2.2.tar.bz2!/lr2.2-public/lib/stan/cmdstan/stan/doc/api/html/agrad_2fwd_2matrix_2determinant_8hpp_source.html]

Stan: src/stan/agrad/fwd/matrix/determinant.hpp Source File


|  |  |
| --- | --- |
|  | Stan  2.5.0  probability, sampling & optimization |


- Main Page
- Related Pages
- Namespaces
- Classes
- Files

- File List
- File Members

All Classes Namespaces Files Functions Variables Typedefs Enumerations Enumerator Friends Macros Pages

- src
- stan
- agrad
- fwd
- matrix

determinant.hpp

Go to the documentation of this file.

1 #ifndef STAN\_\_AGRAD\_\_FWD\_\_MATRIX\_\_DETERMINANT\_HPP

2 #define STAN\_\_AGRAD\_\_FWD\_\_MATRIX\_\_DETERMINANT\_HPP

3

4 #include <vector>

5 #include <boost/math/tools/promotion.hpp>

6 #include <stan/math/matrix/Eigen.hpp>

7 #include <stan/agrad/fwd/fvar.hpp>

8 #include <stan/agrad/fwd/matrix/typedefs.hpp>

9 #include <stan/math/matrix/multiply.hpp>

10 #include <stan/agrad/fwd/matrix/multiply.hpp>

11 #include <stan/math/matrix/inverse.hpp>

12 #include <stan/agrad/fwd/matrix/inverse.hpp>

13 #include <stan/math/error\_handling/matrix/check\_square.hpp>

14

15 namespace stan {

16  namespace agrad {

17

18  template<typename T, int R,int C>

19  inline

20  fvar<T>

21  determinant(const Eigen::Matrix<fvar<T>, R, C>& m) {

22  using stan::math::multiply;

23

24  stan::math::check\_square("determinant(%1%)",m,"m",(double\*)0);

25  Eigen::Matrix<T,R,C> m\_deriv(m.rows(), m.cols());

26  Eigen::Matrix<T,R,C> m\_val(m.rows(), m.cols());

27  Eigen::Matrix<T,R,C> m\_inv(m.rows(), m.cols());

28  fvar<T> result;

29

30  for(size\_type i = 0; i < m.rows(); i++) {

31  for(size\_type j = 0; j < m.cols(); j++) {

32  m\_deriv(i,j) = m(i,j).d\_;

33  m\_val(i,j) = m(i,j).val\_;

34  }

35  }

36

37  m\_inv = stan::math::inverse(m\_val);

38  m\_deriv = multiply(m\_inv, m\_deriv);

39

40  result.val\_ = m\_val.determinant();

41  result.d\_ = result.val\_ \* m\_deriv.trace();

42

43  return result;

44  }

45  }

46 }

47 #endif

inverse.hpp

stan::agrad::fvar

**Definition:** fvar.hpp:13

typedefs.hpp

stan::agrad::multiply

Eigen::Matrix< fvar< T >, R1, C1 > multiply(const Eigen::Matrix< fvar< T >, R1, C1 > &m, const fvar< T > &c)

**Definition:** multiply.hpp:21

stan::agrad::size\_type

Eigen::Matrix< double, Eigen::Dynamic, Eigen::Dynamic >::Index size\_type

**Definition:** typedefs.hpp:14

fvar.hpp

inverse.hpp

Eigen.hpp

stan::math::inverse

Eigen::Matrix< T, Eigen::Dynamic, Eigen::Dynamic > inverse(const Eigen::Matrix< T, Eigen::Dynamic, Eigen::Dynamic > &m)

Returns the inverse of the specified matrix.

**Definition:** inverse.hpp:18

multiply.hpp

stan::agrad::fvar::d\_

T d\_

**Definition:** fvar.hpp:16

stan::math::multiply

boost::enable\_if\_c< boost::is\_arithmetic< T >::value, Eigen::Matrix< double, R, C > >::type multiply(const Eigen::Matrix< double, R, C > &m, T c)

Return specified matrix multiplied by specified scalar.

**Definition:** multiply.hpp:25

stan::agrad::determinant

fvar< T > determinant(const Eigen::Matrix< fvar< T >, R, C > &m)

**Definition:** determinant.hpp:21

multiply.hpp

stan::math::check\_square

bool check\_square(const char \*function, const Eigen::Matrix< T\_y, Eigen::Dynamic, Eigen::Dynamic > &y, const char \*name, T\_result \*result)

Return true if the specified matrix is square.

**Definition:** check\_square.hpp:24

check\_square.hpp


---

[ Stan Home Page ]

*© 2011–2014,
Stan Development Team.*
